# Intelligent Detection and Odor Recognition of Cigarette Packaging Paper Boxes Based on a Homemade Electronic Nose

**DOI:** 10.3390/mi15040458

**Published:** 2024-03-28

**Authors:** Xingguo Wang, Hao Li, Yunlong Wang, Bo Fu, Bin Ai

**Affiliations:** 1 School of Microelectronic and Communication Engineering, Chongqing University, Chongqing 400044, China; 202212021064t@cqu.edu.cn (X.W.); lihaoo@cqu.edu.cn (H.L.); 2 College of Tobacco Science, Henan Agricultural University, Zhengzhou 450002, China; wyl18864788176@163.com

**Keywords:** electronic nose, gas sensor array, cigarette packaging paper, odor detection, machine learning

## Abstract

The printing process of box packaging paper can generate volatile organic compounds, resulting in odors that impact product quality and health. An efficient, objective, and cost-effective detection method is urgently needed. We utilized a self-developed electronic nose system to test four different cigarette packaging paper samples. Employing multivariate statistical methods like Principal Component Analysis (PCA), Linear Discriminant Analysis (LDA), Statistical Quality Control (SQC), and Similarity-based Independent Modeling of Class Analogy (SIMCA), we analyzed and processed the collected data. Comprehensive evaluation and quality control models were constructed to assess sample stability and distinguish odors. Results indicate that our electronic nose system rapidly detects odors and effectively performs quality control. By establishing models for quality stability control, we successfully identified samples with acceptable quality and those with odors. To further validate the system’s performance and extend its applications, we collected two types of cigarette packaging paper samples with odor data. Using data augmentation techniques, we expanded the dataset and achieved an accuracy rate of 0.9938 through classification and discrimination. This highlights the significant potential of our self-developed electronic nose system in recognizing cigarette packaging paper odors and odorous samples.

## 1. Introduction

Annually, the Chinese cigarette industry incurs revenue losses amounting to millions of dollars, due to the rampant production and sale of counterfeit cigarettes [1,2,3,4]. Against this backdrop, the detection of odors in cigarette packaging emerges as a critical issue within the tobacco sector. Recent market research has unveiled odor problems in a particular batch of cigarettes, eliciting considerable concern across the industry. These odors are believed to originate from volatile organic compounds (VOCs) present in the cigarette packaging paper and other related materials, markedly influencing consumer perception and the overall quality of the product. The composition of cigarette packaging paper includes a variety of materials such as base paper, adhesives, coatings, inks, and aluminum foil. Among these, cigarette packaging paper stands out as the most complex and susceptible to odors, attributed to sophisticated printing technologies. However, the incomplete volatilization of inks and solvents during the printing process can lead to odor issues, detrimentally affecting the smoking experience and potentially posing health risks to consumers. Considering the varied and intricate nature of the volatile gas components in cigarette packaging paper, there is a pressing need for the development of efficient, objective, and cost-effective techniques to identify and distinguish volatile odors in cigarette box packaging paper.

Electronic Nose (E-nose) technology simulates the olfactory mechanism of living organisms by utilizing an array of sensors to capture response information from target gas components. Through the application of data processing techniques and intelligent algorithms, E-nose technology facilitates the comprehensive data collection and processing of sample components. This enables the effective detection and identification of gases and odors [5,6]. Unlike traditional chemical analysis instruments, E-nose analysis does not provide qualitative and quantitative results for specific components within the tested sample. Rather, it offers holistic information about the volatile components in the sample, often described as “fingerprint” data. This technology excels in analyzing sample odors that have complex media and components, offering advantages such as rapid detection, cost efficiency, a broad detection range, and the elimination of the need for chemical reagents. In odor analysis applications, E-nose technology enables comprehensive, objective, accurate, and swift odor evaluations [7,8,9]. In their research, Luo Dehan et al. [10] utilized portable E-nose technology to collect and classify aroma signals from various cigarette brands. They employed Artificial Neural Networks (ANNs) for system training, using raw data for model training and extracting features from E-nose-collected data to differentiate between cigarette types. This study strongly supports the application of E-nose technology in the recognition of cigarette brands. Conversely, Zhiyuan Wu et al. [11] developed a portable, cost-effective E-nose system for cigarette brand identification. They applied machine learning algorithms to analyze E-nose data, with their results highlighting significant odor distinctions among different cigarette brands. The Random Forest (RF) algorithm, in particular, achieved the highest accuracy rate of 91.67%, underlining the potential of E-nose technology in distinguishing cigarette brands.

From the literature review [8,12,13,14], we identified the following two primary limitations in existing studies: First, current electronic nose systems necessitate the preconcentration of sample gases to increase gas concentration, which extends the detection time to over 20 min for completing the analysis. Second, the sensor arrays in self-developed portable electronic nose systems are often limited, thereby restricting their ability to comprehensively detect and analyze the various odors present in cigarettes. In contrast, traditional detection methods, such as gas chromatography–mass spectrometry, are hampered by their complex operation and lengthy detection cycles [15]. Our research seeks to address these limitations by proposing a new electronic nose system that features a shorter preconcentration time and a more extensive array of sensors. This will enable the comprehensive detection and analysis of cigarette odors, offering a more efficient solution for cigarette quality control.

To overcome the previously mentioned limitations, we independently developed an advanced electronic nose system. This system serves as a pivotal foundation for our research in constructing models aimed at evaluating the stability and recognizing the odors of cigarette box packaging paper. Our electronic nose system is equipped with a diverse sensor array, comprising more than 40 metal oxide semiconductor sensors and electrochemical sensors. This array is capable of detecting a broad spectrum of gases, thereby facilitating a comprehensive analysis of odors emanating from cigarette boxes. Additionally, our system is designed for efficiency, completing a single detection cycle in just 14 min.

**Figure 1:**
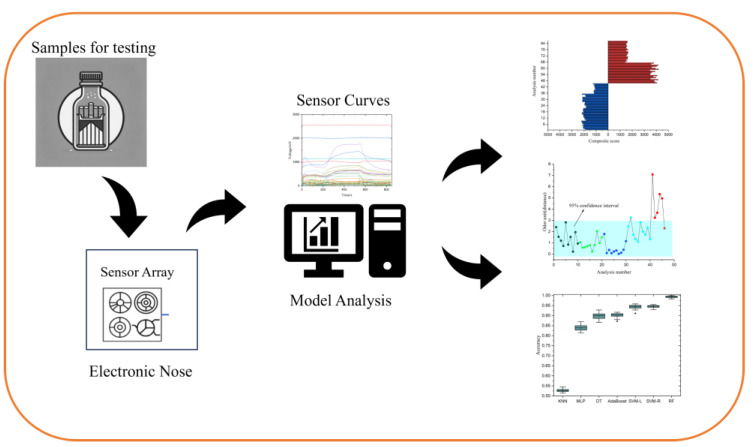
Flowchart of cigarette packaging paper boxes’ detection and analysis based on a homemade electronic nose, including sample detection and data modeling analysis.

In this paper, we conducted multiple tests on samples of cigarette box packaging paper to study the stability of the volatile odors they release. Utilizing Principal Component Analysis (PCA), we developed an extensive model for assessing the stability of the packaging paper. This model enabled us to derive quantitative evaluations of stability. We further applied PCA and Linear Discriminant Analysis (LDA) for dimensionality reduction, followed by the establishment of Statistical Quality Control (SQC) and Soft Independent Modeling of Class Analogy (SIMCA) analyses. Our goal was to innovate a method capable of identifying odors from cigarette box packaging paper and evaluating their quality stability with high precision. Moreover, we validated the effectiveness of our custom-developed electronic nose system in detecting odors from cigarette box packaging. For a comprehensive evaluation, we utilized the following seven distinct algorithms: K-Nearest Neighbors (KNNs), Multilayer Perceptron (MLP), Decision Trees (DTs), Support Vector Machines with a Linear Kernel (SVM-L), Support Vector Machines with a Radial Kernel (SVM-R), Random Forest (RF), and AdaBoost. These algorithms were employed to classify the odor data captured by our electronic nose system from cigarette boxes. This step was crucial in demonstrating the potential of integrating electronic nose technology with machine learning techniques for accurately classifying odors from cigarette box packaging paper, aiming to pioneer a novel classification method for these odors. The specific experimental procedures and outcomes are illustrated in Figure 1.

## 2. Materials and Methods

### 2.1. Materials

Cigarette box packaging paper and finished cigarette boxes (provided by the College of Tobacco Science, Henan Agricultural University), sample testing containers, acetic ethyl acetate solution (serving as the source of odor), and a micro-injector were used in these studies.

Cigarette Box Packaging Paper Sample Preparation Method

1. Preparation of Qualified Samples: Begin by selecting a piece of cigarette packaging paper, weighing approximately 10 g. Carefully fold the paper in such a manner that the printed surface is exposed, enhancing the release of volatile organic compounds. Next, place the folded paper into a sample container bottle. Allow the sample to stand at room temperature, specifically at an indoor temperature of 24 degrees Celsius, facilitating the natural volatilization of the compounds.
2. Preparation of Odor Samples: Following the procedure for preparing qualified samples, select another piece of cigarette packaging paper and fold it accordingly. Place this folded paper into a sample container bottle as well. Utilizing a micro-injector, introduce 3 microliters of an ethyl acetate solution into the bottle to act as the odor source. Let the sample undergo volatilization at room temperature, maintaining an indoor temperature of 24 degrees Celsius.

Finished Cigarette Pack Sample Preparation Method

1. Preparation of Qualified Samples: Begin by carefully opening each box of the finished cigarette samples. Remove the cigarettes, the tin foil, and the cardboard, ensuring the cigarette box remains intact. Subsequently, place this intact cigarette box into a sample container bottle. Allow the box to volatilize at room temperature, set at an indoor temperature of 24 degrees Celsius, to facilitate the natural emission of volatile compounds.
2. Preparation of Odor Samples: Follow the initial steps as in the preparation of qualified samples by removing the cigarettes, tin foil, and cardboard from the packaging box, keeping the cigarette box intact. Place the intact box into a sample container bottle. Then, using a micro-injector, introduce 3 microliters of an ethyl acetate solution into the bottle to serve as the odor source. Permit the sample to volatilize at room temperature, maintaining an indoor temperature of 24 degrees Celsius.

### 2.2. Experimental Setup

We conducted experiments on the following two distinct sample types: cigarette box packaging paper and complete cigarette boxes. To assess the influence of equilibration time on the response curve of our custom-built electronic nose, we performed tests under two varying equilibration periods, 30 min and 5 min. Accordingly, we designated the samples as follows: sample A (cigarette box packaging paper equilibrated for 30 min), sample B (finished cigarette pack equilibrated for 30 min), sample C (cigarette box packaging paper equilibrated for 5 min), and sample D (finished cigarette pack equilibrated for 5 min). Additionally, in line with the previously described “preparation method for odor samples”, we tested two variants of box packaging paper odor samples, each equilibrated for 30 min, and referred to as sample E for simplicity. The total count of samples included 35 for sample A; 20 each for samples B, C, and D; and 6 for sample E.

During the odor data collection from these samples, we meticulously set the operational parameters for the homemade electronic nose. These parameters encompassed baseline acquisition time, post-baseline duration, sample injection period, and the sensor array’s cleaning interval. For the Mass Flow Controller (MFC) flow rate settings, we typically selected an integer value within the 0–500 mL/min range. The default settings were applied across all parameters, including a 2 min air baseline acquisition at a flow rate of 500 mL/min and temperature of 30 °C; a 1 min post-air baseline period at 40 mL/min and 30 °C; a 6 min sample injection at 40 mL/min and a high temperature of 270 °C; followed by a 5 min sensor array cleaning at 500 mL/min and 30 °C.

### 2.3. Data Processing

To minimize unwanted noise and interference in the time-series data from the electronic nose, while still preserving the integrity of original data trends, we initially adopt the moving average method for processing the raw response data [16]. The moving average is a statistical technique designed to smooth out data points by creating a series of averages of different subsets of the full dataset. This approach is particularly effective for time-series data, as it helps to mitigate both short-term fluctuations and long-term variations. The window size for the moving average method is set to 5. Zero-padding is applied to the first data point to ensure smooth consistency and the same method is applied to the last data point to maintain data integrity. The detailed formula for the moving average method is presented in Equation (1). Here, *x*_*t*_ represents the original response data, while *X*_*t*_ denotes the data after moving average processing.

Data normalization is a critical technique for adjusting measurements taken on different scales to a conceptually unified scale. Given that the resistance values captured by each sensor span a broad range, proper normalization of gas sensor data is essential for enhancing the reliability of predictions for future test data, including those that may extend beyond the range of the training dataset. Several normalization methods exist, such as z-score normalization [17], min–max normalization [18], and baseline normalization [19]. While z-score normalization is widely used, it may not effectively handle non-stationary time-series data. Therefore, in this study, we consider min–max normalization. The formula for min–max normalization is depicted in Equation (2), where *X* denotes the sensor data of the time series, max (*X*) and min (*X*) represents the highest value within the data sequence, and the lowest. This method transforms the minimum value of each feature to 0, the maximum value to 1, and all other values to a proportional decimal within the 0 to 1 range.


(1) $$X_{t}=\frac{x_{t-2}+x_{t-1}+x_{t}+x_{t+1}+x_{t+2}}{5}$$

(2) $$X_{\mathrm{normalized}}=\frac{X-\min(X)}{\max(X)-\min(X)}$$

We deployed our custom sensor array to capture odor data from cigarette packaging paper samples, generating corresponding sensor signal curves. These data formed the basis of our cigarette packaging paper sample database. To streamline data complexity, we utilized feature extraction techniques such as Principal Component Analysis (PCA) and Linear Discriminant Analysis (LDA), which provided us with more concise data representations. Following this dimensionality reduction, we applied sophisticated analytical methods, including Statistical Quality Control (SQC) and Soft Independent Modeling of Class Analogy (SIMCA), for the in-depth analysis and processing of the data. We established standardized stability confidence intervals to evaluate the relative stability of the cigarette packaging paper samples, facilitating the classification of quality stability and odor differentiation among the samples. Furthermore, to quantitatively assess the stability of these samples, we developed an extensive evaluation model based on PCA. We then calculated the relative standard deviation (RSD) of the sample data utilizing comprehensive score values, enhancing our understanding of sample stability.

For the classification and recognition of cigarette packaging paper, we employed machine learning algorithms such as AdaBoost, DTs, RF, KNNs, SVM-L, SVM-R, and MLP. Initially, we performed feature extraction on response signals from each sensor, followed by combining these feature levels into individual samples. Multiple rows of combined features formed a feature matrix for subsequent algorithm processing. Given the relatively small dataset, we utilized computer simulation and sequence cross-recombination to augment the dataset. We used 20-fold cross-validation to obtain the average accuracy. We employed grid search to determine the best hyperparameters for the models. Specifically, for KNNs, we set the number of nearest neighbors (n_neighbors) to 10; for DTs, we specified a maximum tree depth (max_depth) of 20; for RF, the number of trees (nTree) was set to 10 and tree depth (max_depth) was set to 10; for SVM-L, we employed a linear kernel function (Kernel) and set gamma to “auto”; for SVC-R, we used a radial basis function kernel (kernel) and a penalty factor C set to 100; for MLP, we configured 100 hidden layer neurons, employed ReLU (Rectified Linear Unit) as the activation function, set the learning rate to 0.001, the maximum iteration count to 200, and used the Adam optimizer for model training; for AdaBoost, we set the number of weak classifiers (n_estimators) to 10 and the learning rate parameter to 0.7.

## 3. Results and Discussion

**Figure 2:**
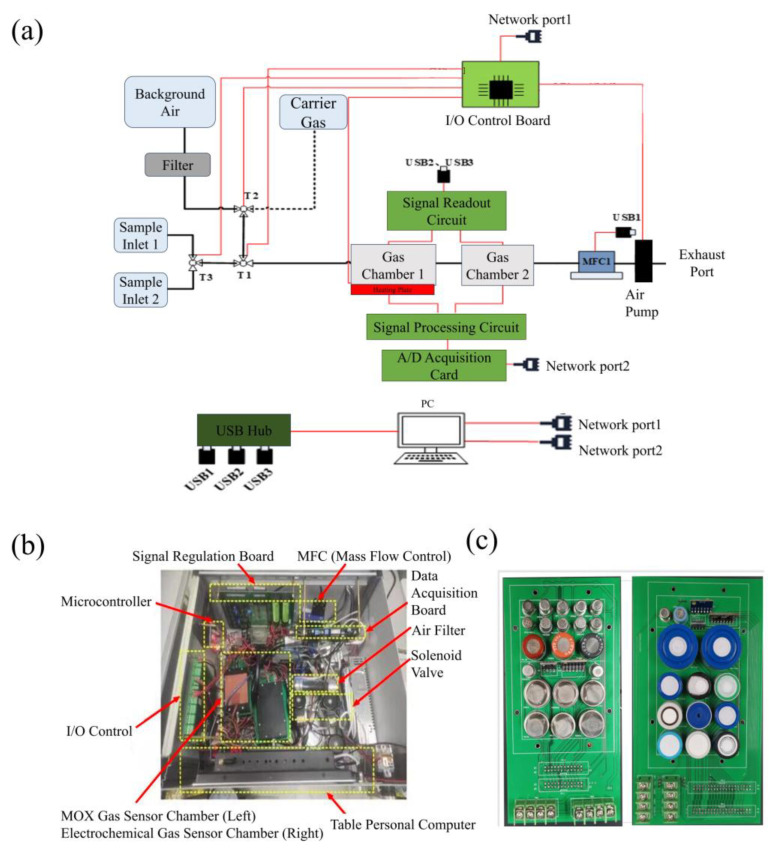
Homemade electronic nose system. (**a**) Schematic diagram of the electronic nose system. (**b**) Real-life internal structure. (**c**) Internal sensor array of the electronic nose.

A structural diagram of the homemade electronic nose system utilized in this study is depicted in Figure 2a. Within the system schematic, solid black lines denote the gas flow path, solid red lines indicate control signals, and dashed black lines represent the standby carrier gas path designed for potential integration with preconcentration systems [20]. Notably, all experiments conducted in this study were executed in a non-preconcentration mode. This means that the detection and analysis of gas samples proceeded without the use of preconcentration techniques to amplify the sensitivity or concentration of the target gases. Figure 2b illustrates an actual view of the homemade electronic nose system’s internal structure, featuring all essential components such as an odor sensor array, a gas sampling unit, a detection unit, and a control unit, along with an optional preconcentration unit and a software upgrade interface [21]. Figure 2c showcases the sensor array within the homemade e-nose, highlighting it as the pivotal element of the system. Our electronic nose is equipped with an array of sensor types, encompassing both metal oxide semiconductor sensors and electrochemical sensors [22]. Metal oxide semiconductor sensors generally function within a temperature range of 200 °C to 500 °C, whereas electrochemical sensors are designed to operate at or around room temperature. To accommodate the diverse temperature requirements of these sensors, our system incorporates two specialized sensor gas chambers. These chambers not only adhere to the operational temperature needs but also enhance the efficiency of gas preconcentration and extend the gas detection limits [20,23].

The software component of our homemade electronic nose system boasts remarkable robustness. Upon setting the appropriate parameters, it can seamlessly collect data on temperature, humidity, pressure, and gas sensor readings from the sensor chambers in real time. This functionality allows users to monitor the progress of experiments with precision, thereby enhancing control over their duration. The system’s capability to instantaneously detect variations in sensor response curves upon the introduction of gases from the samples facilitates the rapid invocation of relevant algorithms for swift sample type identification. This method supersedes traditional manual analysis, significantly increasing the efficiency of sample detection.

Moreover, the homemade electronic nose system integrates a series of commonly used pattern recognition and odor measurement algorithms [12], including Principal Component Analysis (PCA) [24], Linear Discriminant Analysis (LDA) [25], Back-Propagation Artificial Neural Networks (BP-ANNs) [26], Support Vector Machines (SVMs) [27], k-Nearest Neighbors (KNNs) [28], Decision Trees [29], and more. The system includes an automatic calibration algorithm module that effectively addresses sensor drift issues, further improving the accuracy of gas detection and identification [21,30,31,32].

### 3.1. Analysis of the Specificity and Sensitivity of the Sensor on the Sample’s VOCs

**Figure 3:**
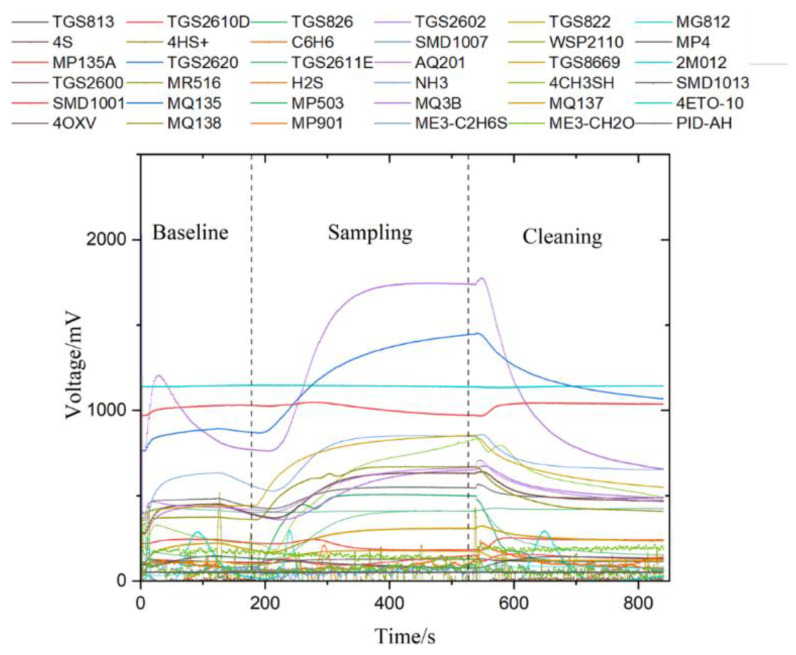
Sensor response curve of sample A, including baseline stage, sampling stage, and sensor cleaning stage.

We utilized a homemade electronic nose to evaluate different types of cigarette box packaging paper samples. As illustrated in Figure 3, we present the sensor response curve for sample A, which is categorized into the following three primary phases: the air baseline stage (3 min), the sampling stage (6 min), and the sensor cleaning stage (5 min), cumulating in a total detection duration of 14 min. During the sampling phase, sensors such as TGS2602, MQ135, TGS2620, MQ137, WSP2110, MQ138, and MQ3B demonstrated a pronounced and noticeable reaction to the odors emitted by cigarette packaging paper. Notably, TGS2602 shows a heightened sensitivity to volatile organic compounds (VOCs) like alcohol and formaldehyde, whereas MQ135 is particularly responsive to hazardous gases including carbon monoxide, nitrogen oxides, and ammonia. Furthermore, MQ138 and MQ3B sensors exhibit acute sensitivity to VOCs such as benzene, alkanes, and alcohol. The distinctive responses from these sensors highlight the presence of substantial quantities of these VOCs in the odors emanating from the cigarette packaging paper, affirming the precision and specificity of our electronic nose system in identifying and differentiating between various types of cigarette packaging papers, each marked by unique response traits. For additional response feature data concerning other samples, refer to the Supplementary Materials, Section S1.

We further performed a preliminary comparative analysis, showcasing the sensor response curves for cigarette packaging paper samples with a static time of 30 min, samples of finished product packaging boxes, and their respective odor samples, detailed in the Supplementary Materials, as depicted in Figure S2. Specifically, Figure S2a,b illustrate the sensor responses for qualified and odor samples of cigarette box packaging paper, respectively, while Figure S2c,d display the responses for qualified and odor samples of finished product packaging boxes. Notably, the sensor response to odor samples is significantly more pronounced than that of the qualified samples, signifying the richness of odor components present. This comparative analysis of the response curves offers initial insights into the olfactory distinctions between cigarette packaging paper, finished product packaging boxes, and their odor samples, with the sensor responses serving as a solid basis for data support.

### 3.2. Feature Selection and Validity Verification

**Figure 4:**
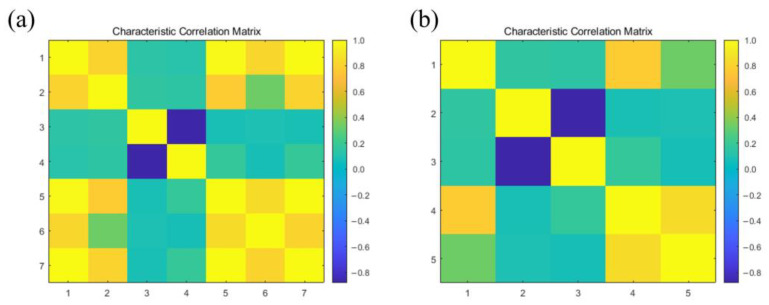
Characteristic correlation matrix. (**a**) Correlation matrix of preferred seven features. (**b**) Correlation matrix of the five selected features.

Initially, we selected the following features from seven candidate features [33]: 1. maximum value during sampling phase, 2. difference between maximum value during sampling phase and baseline mean, 3. maximum slope during sampling phase, 4. minimum slope during sampling phase, 5. area under the curve during sampling phase, 6. steady-state value during sensor cleaning phase, and 7. mean value of the last three minutes of the sampling phase. Subsequently, we conducted feature correlation analysis, as shown in Figure 4a, revealing strong correlations between feature 1 and features 2, 5, 6, and 7. Additionally, feature 5 exhibited strong correlations with features 1, 2, 6, and 7. Following this, we performed Principal Component Analysis on the feature data to calculate the contribution rates of each feature. The contribution rates were found to be as follows: 0.0002, 0.6218, 0.0103, 0.0015, 0.0001, 0.2692, and 0.0968 for features 1 to 7, respectively. Owing to the low contribution rates of features 1 and 5, we opted to exclude these two features and retained the remaining five for further modeling analysis and machine learning classification. The correlation matrix for the selected five features is presented in Figure 4b and a detailed representation of the features is provided in Table 1.

Due to the significant redundancy in the individual signal spectra of the samples, which were represented by an 840 × 36 matrix, managing such extensive data proved challenging for the effective analysis and processing of odor data from the packaging paper. Therefore, we applied feature extraction and sensor array optimization to the signal spectra [19,34]. Details of sensor array optimization can be found in the Supplementary Materials.

### 3.3. Evaluation of the Odor Quality Stability of Cigarette Box Packaging Paper

This study utilized a homemade electronic nose system to perform odor testing on cigarette packaging paper and finished cigarette boxes, collecting data for samples A, B, C, and D at resting times of both 30 min and 5 min. We explored the volatility of odors across each sample category and confirmed the stability of our electronic nose system. To achieve quantitative analysis, we constructed a comprehensive evaluation model for the stability of packaging paper using Principal Component Analysis (PCA). Additionally, we calculated the relative standard deviation (RSD) for each sample as a means to assess the stability level of cigarette packaging paper, thereby providing a systematic approach to quantifying odor stability.

**Figure 5:**
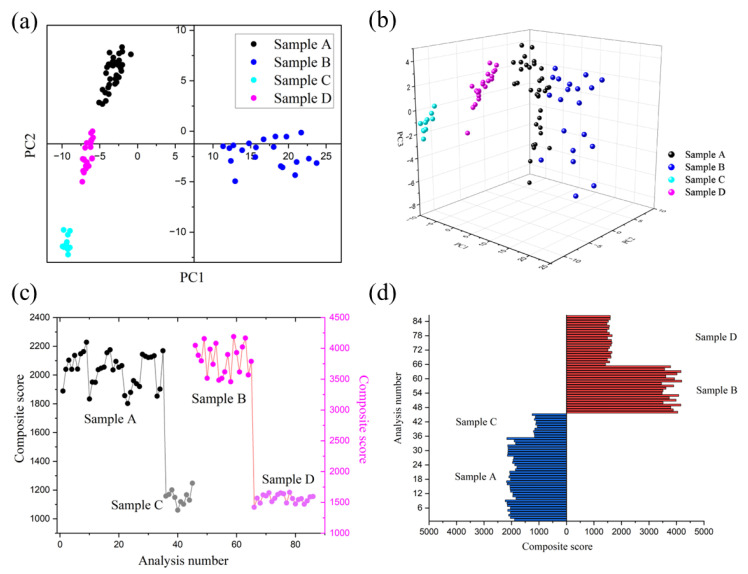
Evaluation of odor quality stability of cigarette box packaging paper. (**a**) PCA two-dimensional scatter plot. (**b**) PCA three-dimensional scatter plot. (**c**) Stability fluctuation chart. (**d**) Comprehensive score bar chart.

Principal Component Analysis (PCA) is a commonly used multivariate statistical analysis method, often used for dimensionality reduction and data visualization [35]. Its main objective is to convert high-dimensional data into a lower-dimensional form by identifying the primary directions of variation (principal components) within the data, thereby retaining as much information from the original dataset as possible. Using the PCA algorithm, we reduced the sensor signal spectra of four different categories of samples to two-dimensional and three-dimensional representations, generating scatter plots, as shown in Figure 5a,b. In Figure 5a, the first principal component distinguishes sample B from the other three categories, while the separation of samples A, C, and D primarily relies on the second principal component. This suggests that there are unique features or characteristics present in sample B that differentiate it from samples A, C, and D. Conversely, the separation of samples A, C, and D appears to be primarily driven by variations along the second principal component. In Figure 5b, the three-dimensional scatter plot provides a clearer depiction of the differences and similarities among the samples across multiple dimensions. we gain additional insight into the relationships between the samples that may not be fully captured in the two-dimensional representation. In these plots, data points from the same category were closely clustered together, while data points from different categories were well separated, achieving good classification and discrimination effects.

For sample B, the scatter plot exhibited considerable dispersion, signifying notable variability within this category’s sample data and suggesting lower stability. Therefore, during the PCA process, we selected principal components with a cumulative contribution rate of 90% and weighted them according to their contribution rates. This allowed us to establish a comprehensive evaluation model for the stability of packaging paper based on Principal Component Analysis and calculate the composite score for each batch of samples.

The stability fluctuation chart, depicted in Figure 5c, serves to illustrate the stability levels of the samples. Smaller fluctuations in stability represent more stable sample data. In the chart, samples A and B, which rested for 30 min, exhibited greater fluctuations, primarily due to their longer resting times, leading to richer volatile odor components. This pattern was further corroborated in Figure 5d, where these samples registered higher composite scores. Furthermore, under the same conditions, finished cigarette box samples generally had higher composite scores than cigarette packaging paper samples, indicating that finished cigarette box samples had more diverse odors, possibly including the aroma of cigarettes or other odors. This finding aligns with real-world observations. To quantitatively assess the stability of the various sample types, we calculated the relative standard deviation (RSD) for the composite scores of each sample, as detailed in Table 2. The outcomes of these RSD calculations align with the observations and analysis presented in Figure 5c,d, reinforcing the conclusions drawn from the study.

### 3.4. Box Packaging Paper Quality Stability Discrimination

In this study, we employed the Statistical Quality Control (SQC) [36] and the Soft Independent Modeling of Class Analogy (SIMCA) algorithms [9,37] to analyze the stability of data within sample groups and between sample groups. We conducted an intra-group stability discriminant analysis for data from stationary 30 min cigarette packaging paper samples (sample A) and finished cigarette packs (sample B). Furthermore, for inter-group stability analysis, we selected ten datasets each from Samples A, B, C, and D, along with six sets of odor data (three each from samples A and B).

SQC is a widely adopted technique for evaluating the stability of sample quality, based on the assumption that the sample data follow a normal distribution. It involves calculating the 95% confidence interval for the data of standard cigarette packaging paper. During the assessment of quality stability, we calculate the confidence level for any unknown samples and compare it with the 95% confidence interval of the standard sample data to determine stability. Samples within this confidence interval are determined to have stable quality.

**Figure 6:**
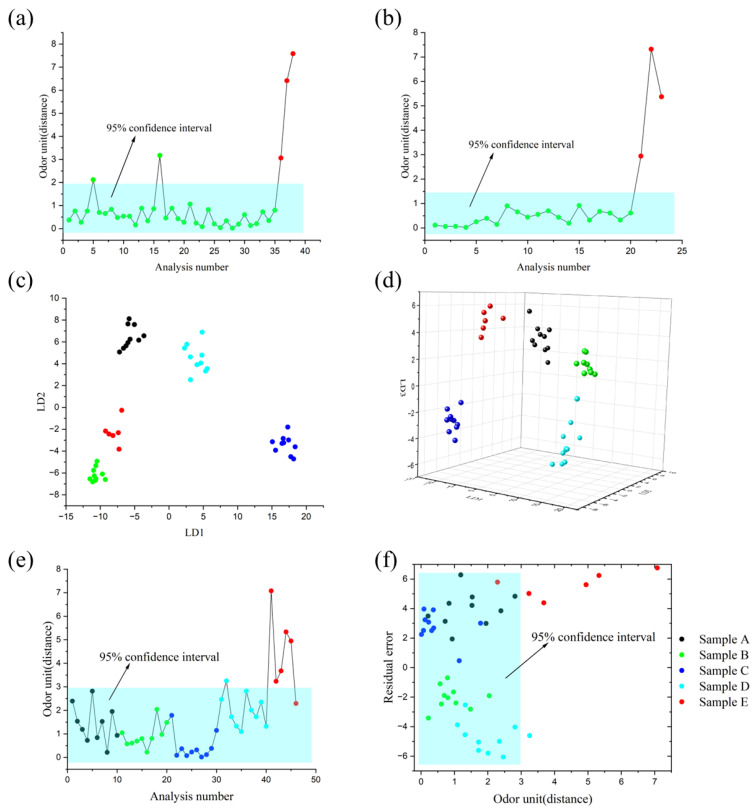
Analysis of odor quality stability and odor discrimination in cigarette box packaging paper. (**a**) SQC analysis of cigarette box packaging paper. (**b**) SQC analysis of finished cigarette boxes. (**c**) Two-dimensional scatter plot of four sample types in LDA. (**d**) Three-dimensional scatter plot of four sample types in LDA. (**e**) Between-class SQC analysis of four sample types. (**f**) SIMCA analysis of four sample types.

In this part of the work, we first established an SQC model using 35 sets of standard qualified sample A data and calculated the 95% confidence interval for standard sample data. We then compared three sets of odor samples with this model. The results, as shown in Figure 6a, indicate that most of the qualified samples are within the confidence interval, while the odor samples are outside the confidence interval. Notably, sample 5 and sample 16 were classified as odor samples, which could be attributed to potential experimental system inaccuracies. These inaccuracies might stem from a variety of sources including equipment malfunctions, calibration discrepancies, environmental variability, or human error during the handling of samples or data recording. Similarly, the processing results for sample B are shown in Figure 6b. The test results are generally consistent with the actual situation. By utilizing the SQC odor monitoring model established using electronic nose detection data, we can quickly determine the odor quality of unknown samples, thus effectively conducting quality control. This validates the feasibility of electronic nose technology in the detection of entry odors in cigarette packaging materials.

Furthermore, we implemented Linear Discriminant Analysis (LDA) for dimensionality reduction on ten datasets from each of the four sample categories, and six datasets from odor samples, producing condensed scatter plots as illustrated in Figure 6c,d. Subsequently, we constructed inter-class SQC models, as illustrated in Figure 6e. The results indicated that the majority of data points from the four categories of samples fell within the 95% confidence interval. However, sample 32 was found outside the control limits and was identified as an odor sample due to its deviation from the category center, as visually represented in Figure 6d. This deviation led to its misclassification as an outlier. Meanwhile, the 46th sample exhibited odor characteristics similar to those of qualified samples and was, therefore, classified as such by the model. Furthermore, Figure 6f reveals that the data points of the 46th sample fell within the data point range of sample A, suggesting that this data shared similar odor characteristics with sample A.

Furthermore, we utilized the Soft Independent Modeling of Class Analogy (SIMCA) algorithm to analyze samples A through E. The SIMCA algorithm selects relevant principal components and projects them into a subspace, followed by fitting and classifying the samples based on thresholds [37]. As shown in Figure 6f, the SIMCA analysis results exhibited some similarity to the SQC results (seen in Figure 6e). Most data points from samples A to D fell within the 95% confidence interval (colored box region), while data points from other samples predominantly lay outside this region. This suggests that the electronic nose system considered samples A to E to possess similar odor characteristics, which aligns with the actual situation. The response spectrum of our homemade electronic nose system consistently matched the actual odors emanating from cigarette packaging paper, showcasing its ability to discern subtle odor differences with a greater objectivity and accuracy than the human nose. This underscores the effectiveness of electronic nose technology in monitoring and ensuring the quality stability of cigarette packaging paper and in identifying distinct odors.

### 3.5. Cigarette Box Packaging Paper Odor Classification

**Figure 7:**
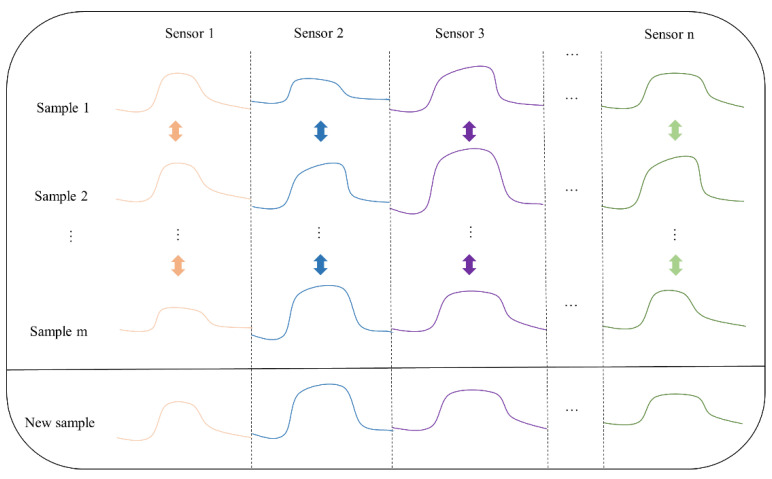
Schematic diagram of sequence crossover recombination. It shows the crossover recombination of sequence data between the same sensors from samples of the same category.

In order to identify and classify different samples of boxed packaging paper as well as samples with unusual odors, we constructed a dataset comprising 40 sets of data for boxed cigarette packaging paper samples and 40 sets for finished cigarette box samples. To broaden the dataset’s diversity and scope, we implemented two data augmentation techniques, as follows: computer simulation and sequence cross-recombination. Specifically, we used these two methods separately to expand the original 40 sets of data, resulting in two distinct categories of datasets, each containing 120 sets of data. For the cases where there was a scarcity of samples for unusual odors, we only utilized the sequence cross-recombination method. In total, we had 360 sets of data available for machine learning training and testing. A schematic diagram of sequence cross-recombination is illustrated in Figure 7. Detailed descriptions and methods regarding computer simulation and sequence cross-recombination can be found in the Supplementary Materials.

**Figure 8:**
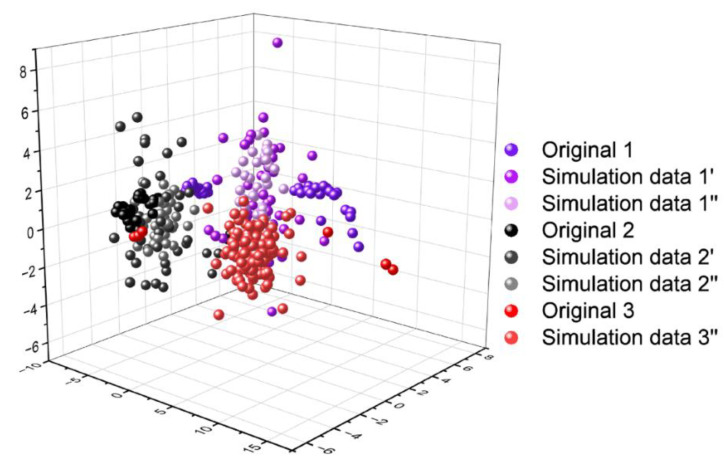
Visualization of three types of sample data. The data for each of the three sample types have been augmented to 120 for subsequent machine learning pattern recognition.

We utilized Principal Component Analysis (PCA) to analyze the dataset, as illustrated in Figure 8. In the figure, the purple data points represent the original data and simulated data for boxed cigarette packaging paper samples, while the black data points represent the data for finished packaging box samples and their simulated counterparts. The red data points denote unusual odor data and their simulated counterparts. Through the application of PCA, we gained the ability to visualize the dataset more effectively, enabling a deeper exploration of the interconnections among the various data points.

Next, we employed seven different classification algorithms to train and test the samples, including K-Nearest Neighbors (KNNs), Multilayer Perceptron (MLP), Decision Trees (DTs), AdaBoost, Support Vector Machines with Linear Kernel (SVM-L), Support Vector Machines with Radial Kernel (SVM-R), and Random Forest (RF). To evaluate the performance of our proposed classification models, we implemented a rigorous evaluation strategy employing a 20 times 10-fold cross-validation method. In each cross-validation iteration, we divided the dataset into ten equal subsets, then iteratively used nine subsets for training and one subset for testing. This process was repeated 20 times, using different combinations of training and testing sets each time. Ultimately, we computed the average accuracy, precision, recall, and F1 score of each model across all testing sets to assess its performance.

**Figure 9:**
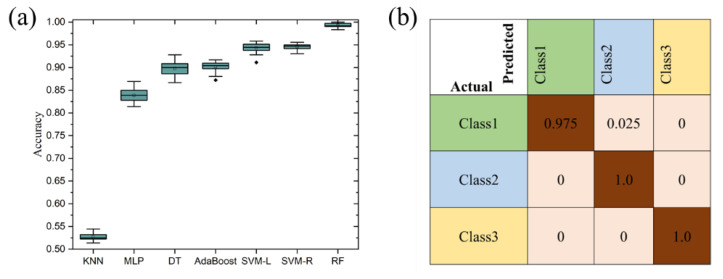
Machine learning classification results. (**a**) Boxplot of accuracy. (**b**) Confusion matrix.

Table 3 displays the performance metrics of different classification algorithms. Regarding average accuracy, Random Forest (RF) exhibited the best performance, reaching 0.9938, followed by Support Vector Machines with Radial Kernel (SVM-R) and Support Vector Machines with Linear Kernel (SVM-L). The accuracy boxplots of the seven algorithms are shown in Figure 9a. For Precision, Recall, and F1 scores, RF, similarly, performed remarkably well, followed by SVM-R and SVM-L. Decision Trees (DTs) and AdaBoost also demonstrated good performance, while K-Nearest Neighbors (KNNs) displayed lower performance. Overall, the Random Forest model demonstrated the best comprehensive performance on this dataset, with its high accuracy, recall, and F1 score indicating its effectiveness in classification tasks. These results provide valuable insights for selecting appropriate machine learning models to achieve effective classification of cigarette packaging paper odors.

The confusion matrix for the DTs algorithm used in the classification of cigarette box packaging paper odors is shown in Figure 9b. This confusion matrix provides a detailed view of the DTs algorithm’s classification accuracy. For cigarette box packaging paper samples, the DTs algorithm correctly classified 97.5% of the samples, misclassifying 2.50% of the samples as finished cigarette boxes. For finished cigarette box samples and odor samples, all of them were identified and classified correctly.

Overall, the various classification algorithms demonstrated differing levels of precision, with the DTs algorithm emerging as the most accurate. These results indicate that by using suitable machine learning algorithms, we can effectively differentiate cigarette box packaging paper types and discern odors. The superior performance of the DTs algorithm in this task underscores the importance of PCA-optimized sensor arrays in improving classification accuracy [19,34] (See Section S3 in the Supplementary Materials for more details).

## 4. Conclusions

In summary, this study has made significant advancements in the analysis of odor characteristics in cigarette packaging paper through the development of a homemade electronic nose system and intelligent algorithms. We have successfully established comprehensive evaluation and quality control models, facilitating the rapid detection of sample odors and enabling effective quality control. Therefore, the proposed electronic nose system and analysis methods are not only suitable for large-scale applications but are also characterized by low cost, high speed, and high accuracy. They can be applied to assess the quality stability and odor discrimination of packaging paper products, which is of paramount importance for enhancing the quality and accuracy of tobacco industry packaging paper products.

However, this work remains exploratory in nature. To comprehensively drive quality management and production efficiency in the tobacco industry, we identify key areas for future research. Firstly, further optimization of the electronic nose system’s performance is essential to enhance its ability to recognize complex odors. This involves incorporating advanced sensor technologies, refining pattern recognition algorithms, and expanding the scale of the sample database to handle a broader range of odor variations. Secondly, there is a need to broaden the application range of electronic nose technology within the tobacco industry. Beyond controlling odors in cigarette packaging paper, the technology can be extended to assess other aspects of tobacco quality, such as the evaluation and analysis of the tobacco itself, achieving comprehensive quality monitoring across the industry chain. Lastly, future research should focus on developing green and environmentally friendly production technologies. By combining electronic nose technology for odor monitoring in the tobacco industry with other eco-friendly measures, we can promote a more sustainable and environmentally conscious production process, providing crucial support for the industry’s sustainable development.

## Figures and Tables

**Table 1:** Selected features.

Feature Number	Source
1	The 3 min steady-state average value after the sampling stage.
2	the maximum value at the sampling stage minus the baseline value.
3	Slope vector during the sampling stage: *K* = $\left(y_{1}-y_{2}\right)/\left(x_{1}-x_{2}\right),$ |$x_{1}-x_{2}$| = 5, the minimum value of K.
4	Slope vector during the sampling stage: *K* = $\left(y_{1}-y_{2}\right)/\left(x_{1}-x_{2}\right),$ |$x_{1}-x_{2}$| = 5, the maximum value of K.
5	The steady-state average of the response during the cleaning phase.

**Table 2:** RSD values.

Sample	RSD
A	0.0566
B	0.0649
C	0.0455
D	0.0440

**Table 3:** Performance Comparison of Various Classification Algorithms.

Classification Algorithm	Accuracy	Precision	Recall	F1
KNNs	0.5277	0.5268	0.5261	0.4785
MLP	0.8472	0.8502	0.8473	0.8420
DTs	0.8952	0.8964	0.8954	0.8907
AdaBoost	0.9016	0.9025	0.9013	0.8975
SVM-L	0.9413	0.9424	0.9413	0.9393
SVM-R	0.9452	0.9463	0.9464	0.9439
RF	0.9938	0.9939	0.9936	0.9934

## Data Availability

The data supporting the main findings of this study are available from the corresponding authors upon reasonable request.

## Supplementary Materials

The following supporting information can be downloaded at: https://www.mdpi.com/article/10.3390/mi15040458/s1. Figure S1: Response of the homemade E-nose test on sample A. Figure S2: Comparison of sensor response curves. Figure S3: Accuracy of DT algorithm with different sensor combinations. Table S1: Sensors used in the homemade E-nose. Table S2: Sensor Information and Their Contribution Rate.
