# Supplementary material for: Intelligent Detection and Odor Recognition of Cigarette Packaging Paper Boxes Based on a Homemade Electronic Nose
Source: Micromachines (Basel). 2024 Mar 28;15(4):458. doi: 10.3390/mi15040458 (PMC11052458; doi:10.3390/mi15040458)
Supplement: Supplementary file 1 [file micromachines-15-00458-s001.zip › micromachines-2932352-Supplementary Material.pdf]

Supporting Information

Intelligent Detection and Odor Recognition of Cigarette Packaging Paper Boxes Based on Homemade Electronic Nose

Xingguo Wang[1], Hao Li[1], Yunlong Wang[2], Bo Fu[2]*, and Bin Ai[1]*

[1]School of Microelectronic and Communication Engineering, Chongqing University, Chongqing 400044, China

[2]College of Tobacco Science, Henan Agricultural University, 63 Nongye Road, Zhengzhou 450002, China

*Correspondence: B.A., binai@cqu.edu.cn; B.F., fubo@henau.edu.cn

Section S1. Response of the *E*-nose test

We present the sensor response curve of cigarette packaging paper samples with a static time of 30 minutes, as shown in Figure S1. This curve can be divided into three main phases: the air baseline phase (used for measuring air), the sampling phase (used for measuring the target gas), and the cleaning phase (used for sensor cleaning). During the sampling phase, sensors such as TGS2602, MQ135, TGS2620, MQ137, WSP2110, MQ138, MQ3B, etc., exhibit a strong response to the odor of cigarette packaging paper. All sensor information is shown in Table S1.

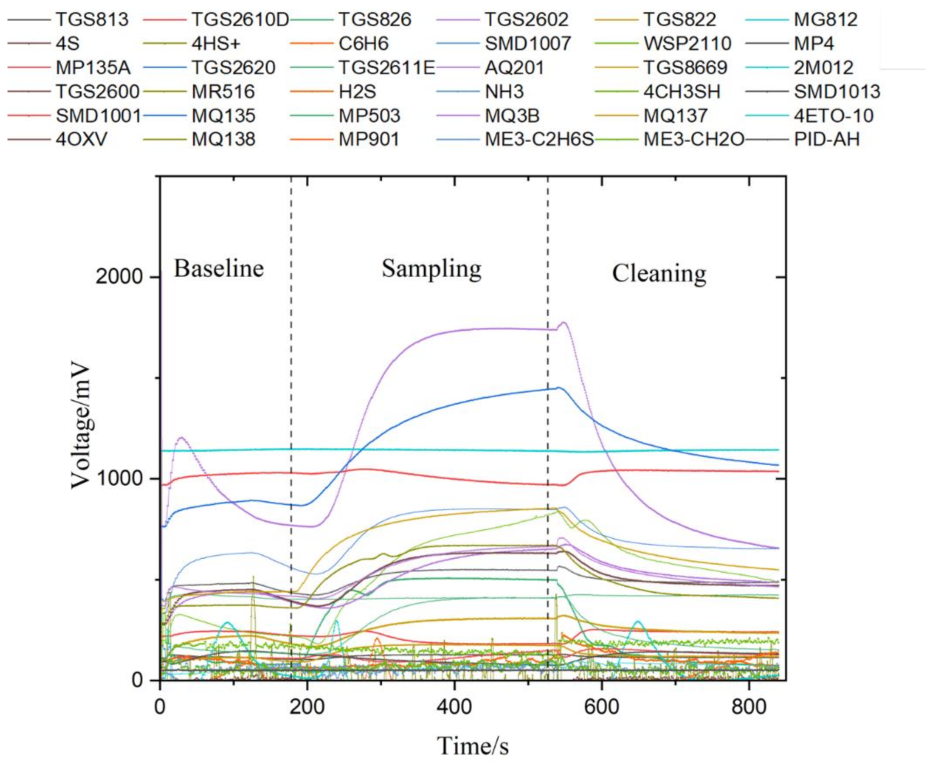

Figure S1. Response of the homemade *E*-nose test on sample A.

Table S1. Sensors used in the homemade *E*-nose.

Sensor Name	Detectable Gas	Range and Precision (PPM)	Sensor Type
TGS813	Methane, Propane, Ethanol, Butane, Isobutane, Hydrogen, and other flammable gases	500 - 1000	Metal oxide
TGS2610D	Volatile organic compounds, Ammonia, Hydrogen sulfide with high sensitivity	1 - 10	Metal oxide
MS1100	Formaldehyde, Toluene, and other organic gases	0 - 1000	Metal oxide
TGS826	Nitrogen compounds (ammonia, amine, etc.), alcohols (ethanol, etc.), hydrocarbons (methane, butane, etc.)	30 - 300	Metal oxide
TGS2602	Toluene, ethanol, H_2S, NH_3, H_2, etc.	1 - 30	Metal oxide
TGS822	Alcohol	50 - 5000	Metal oxide
MG812	Carbon dioxide	0 - 10000	Metal oxide
4HS+	Hydrogen sulfide	0 - 100	Electrochemical
C6H6	Benzene	0 - 100	Electrochemical
SMD1007	Hydrogen sulfide	0 - 3	MEMS
WSP2110	Toluene, Benzene, Formaldehyde and other organic gases with high sensitivity	1 - 50	Metal oxide
MP4	Methane, Natural gas, Biogas	300 - 10000	Metal oxide
MP135A	Alcohol, Smoke	10 - 1000	Metal oxide
TGS2620	Alcohol	50 - 5000	Metal oxide
TGS2611E	Methane	500 - 10000	Metal oxide
TGS8669	Acetone, Toluene, Benzene, etc.	1 - 500	Metal oxide
MR516	Natural gas, Liquefied gas, coal gas, and other flammable gases and various liquid vapors	0 - 1000	Electrochemical
H2S	Hydrogen sulfide	0 - 50	Electrochemical

NH3	Ammonia	0 - 50	Electrochemical
4CH3SH	Methyl mercaptan	0 - 10	Electrochemical
SMD1013	Alcohol, Toluene, Acetone, Formaldehyde, and other volatile organic gases	0 - 100	MEMS
SMD1001	Formaldehyde	0 - 3	MEMS
MQ135	Sensitivity to ammonia, sulfide, benzene series and steam is high	10 - 1000	Metal oxide
MP503	Alcohol, Smoke, Isobutane	1 - 1000	Metal oxide
MQ3B	Alcohol vapor	10 - 1000	Metal oxide
MQ137	Ammonia	5 - 500	Metal oxide
4ETO-10	Epoxyethane	0 - 10	Electrochemical
4OXV	Oxygen	0 - 10	Electrochemical
MQ138	Toluene, Acetone, Ethanol, Hydrogen	5 - 500	Metal oxide
MP901	Alcohol, Smoke, Formaldehyde, Toluene, Benzene, Acetone, Lighter gas, Paint	1 - 50	Metal oxide
ME3-C2H6S	Dimethyl disulfide	0 - 100	Electrochemical
ME3-CH2O	Formaldehyde	0 - 10	Electrochemical
PID-AH	Volatile organic compounds and other toxic gases	0 - 40	Photoionization

We also conducted a preliminary comparative analysis, presenting sensor response curves for cigarette packaging paper samples with a static time of 30 minutes, finished product packaging box samples, and their odor samples, as shown in Figure S2. In Figure S2, (a) and (b) represent qualified samples and odor samples of cigarette box packaging paper, while (c) and (d) represent qualified samples and odor samples of finished product packaging boxes. It is worth noting that the sensor response of the odor samples is significantly higher than that of the qualified samples, indicating the presence of rich odor components. Through the comparative analysis of these response curves, we can gain initial insights into the olfactory differences between cigarette packaging paper, finished product packaging box samples, and their odor samples, with the sensor responses providing robust data support.

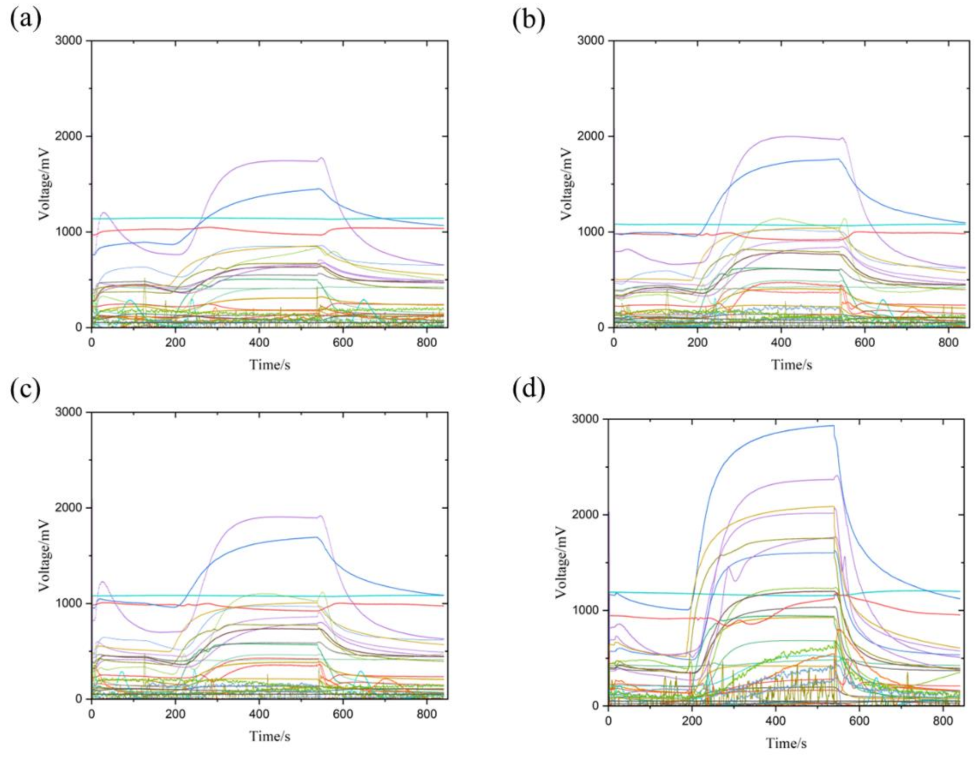

Figure S2. Comparison of sensor response curves. (a) Cigarette box packaging paper with qualified samples. (b) Cigarette box packaging paper with odor samples. (c) Finished product packaging box with qualified samples. (d) Finished product packaging box with odor samples.

Section S2. Data Augmentation Methods

In this study, we employed two primary data augmentation methods for experimentation:

Computer Simulation: Initially, we conducted computer simulations to replicate the distribution characteristics of the original data. This involved generating 40 sets of new data with identical distribution characteristics, using the mean and standard deviation of the original detection data. The objective of this step was to acquire additional data for analysis and comparison. By generating new data with similar

statistical features, we aimed to gain a better understanding and exploration of the nature of sensor responses.

Sequence Cross-Recombination: In this step, we concatenated the sensor response curves into one-dimensional curves and extracted sub-sequences or segments of the same length from them. Subsequently, we exchanged and recombined these sub-sequences with corresponding positions in another curve, thus generating new time sequences of the same length. This process aided in studying the characteristics of sensor response curves and potentially revealed interesting data patterns.

Furthermore, during the generation of new data through computer simulations, we introduced noise, which could be controlled by a noise coefficient. This step aimed to more accurately simulate system noise during electronic nose odor detection in real environments, as such detection is often subject to various sources of interference. By controlling the noise coefficient, we were able to generate data with different noise levels, allowing for a more comprehensive assessment of the performance and robustness of the sensor system.

Section S3. Sensor Array Optimization Based on PCA

We obtained the contribution of each sensor to the principal components through Principal Component Analysis (PCA), and arranged the contribution rates in descending order, as shown in Table S1. Based on this ranking, we determined the number of sensor combinations and selected the top n sensors in order of their contribution rates. For example, if we decide to choose 10 sensors as a combination,

we would select the top 10 sensors in the ranking.

Table S2. Sensor Information and Their Contribution Rate

**Sensor**	**Type**	**Serial number in the array**	**Contribution**
TGS826	MOS	sensor 4	5.410268
AQ201	MOS	sensor 17	5.255676
SMD1007	MEMS	sensor 24	5.24861
TGS813	MOS	sensor 1	5.244931
SMD1013	MEMS	sensor 25	5.24367
MQ3B	MOS	sensor 29	5.113129
ME3-C2H6S	Electrochemical	sensor 35	5.105631
TGS2620	MOS	sensor 15	5.068759
ME4-H2S	Electrochemical	sensor 22	5.067855
4S	Electrochemical	sensor 8	5.03975
MQ135	MOS	sensor 27	5.013944
TGS2600	MOS	sensor 20	5.011211
TGS822	MOS	sensor 6	4.981767
PID-AH	Photo-ionization	sensor 37	4.940078
ME3-CH2O	Electrochemical	sensor 36	4.936504
SMD1007	MEMS	sensor 11	4.903636
4HS+	Electrochemical	sensor 9	4.896518
4OXV	Electrochemical	sensor 32	4.856149
MP503	MOS	sensor 28	4.852512
WSP2110	MOS	sensor 12	4.828378
MQ138	MOS	sensor 33	4.824979
ME4-C6H6	Electrochemical	sensor 10	4.813948
MP135A	MOS	sensor 14	4.806813
SMD1001	MEMS	sensor 26	4.805309

TGS2610D	MOS	sensor 2	4.800094
TGS2602	MOS	sensor 5	4.774675
4ETO-10	Electrochemical	sensor 31	4.771339
MS1100	MOS	sensor 3	4.764009
TGS2611E	MOS	sensor 16	4.758671
NH3-3E100SE	Electrochemical	sensor 23	4.747919
TGS8669	MOS	sensor 18	4.685646
MP4	MOS	sensor 13	4.665699
MR516	Hot line	sensor 21	4.649595
MQ137	MOS	sensor 30	4.565184
2M012	Semi-conductors	sensor 19	4.539368
MP901	MOS	sensor 34	4.487542
MG812	Soild electrolytes	sensor 7	4.465633

Next, we conducted a traversal of the number of different sensor combinations and evaluated the recognition accuracy of the Decision Tree (DT) algorithm using the sensor responses from each combination. The accuracy values we obtained are shown in Figure S3. By optimizing the sensor array using Principal Component Analysis (PCA), we found that when selecting the top 7 sensors with the highest contribution rate, the discrimination accuracy of the DT algorithm improved to 0.9762. When 20 sensors were selected, the recognition accuracy reached its highest level.

This result indicates that through the PCA sensor array optimization algorithm, we can reduce the number of sensors without significantly compromising recognition accuracy, thus optimizing the sensor array. This is of great significance for improving the efficiency and performance of odor recognition systems. Furthermore, this optimization method also helps reduce costs, enhance system scalability, and reduce

the complexity of data processing and computation. Therefore, by carefully selecting sensor combinations, we can more effectively utilize sensor resources without sacrificing recognition performance.

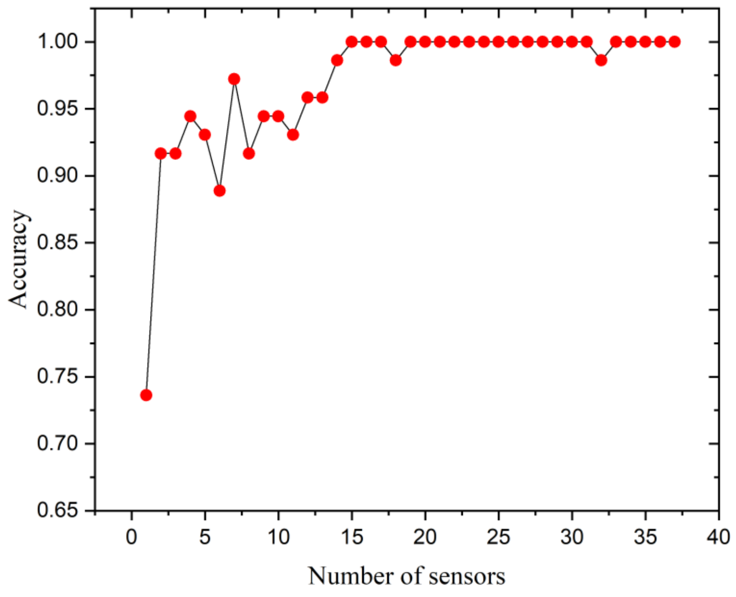

Figure S3. Accuracy of DT algorithm with different sensor combinations
